# Corneal morphological changes after small incision lenticule extraction for myopic anisometropia

**DOI:** 10.3389/fmed.2022.977586

**Published:** 2022-08-24

**Authors:** Lu Zhu, Yan Ji, Xin Yang, Xiaorong Lu, Qiong Wu, Qing Wang, Jiuyi Xia, Meng Li, Ke Hu, Wenjuan Wan

**Affiliations:** Department of Ophthalmology, The First Affiliated Hospital of Chongqing Medical University, Chongqing Key Laboratory of Ophthalmology and Chongqing Eye Institute, Chongqing, China

**Keywords:** refractive surgery, small incision lenticule extraction (SMILE), myopic anisometropia, higher-order aberrations (HOA), posterior corneal elevation (PCE)

## Abstract

**Purpose:**

This research aims to study the corneal morphological changes in adult patients with myopic anisometropia after small incision lenticule extraction (SMILE) and the safety, efficacy, and predictability of clinical outcomes.

**Methods:**

This was a prospective cohort study. Patients with myopic anisometropia [refractive difference >2.0 diopters (D)] were included in this study who underwent SMILE at our hospital from September 2019 to March 2021. For the two eyes of each patient, the one with higher myopia was defined as group A, and the fellow eye was group B. The follow-up time points were set as 1 week, 1 month, 3 months, and 6 months after the surgery. The data collected were uncorrected and best-corrected distance visual acuity (UDVA and CDVA), spherical equivalent (SE), efficacy and safety indexes, posterior corneal elevation (PCE), anterior and posterior corneal radius of curvature in the 3 mm area at the center of the thinnest point of the cornea (ARC and PRC), and higher-order aberrations (HOAs).

**Results:**

The study included 36 patients (72 eyes), and the mean age was 25.2 ± 6.4 years. The preoperative SEs were −6.45 ± 1.25 D in group A and −3.76 ± 1.29 D in group B. Six months after surgery, the SEs in groups A and B were −0.09 ± 0.50 D and 0.07 ± 0.47 (*P* = 0.059), respectively. The efficacy indexes were 1.06 ± 0.16 in group A and 1.07 ± 0.14 in group B (*P* = 0.750). The safety indexes were 1.08 ± 0.14 in group A and 1.12 ± 0.15 in group B (*P* = 0.173). The PCE was significantly reduced at 6 months after surgery in pagebreak both groups (*P* < 0.05). The ARC was significantly higher than before the surgery (*P* < 0.05) in the two groups. The two groups showed significant increases in total HOAs, coma 90°, and spherical aberrations (*P* < 0.05).

**Conclusion:**

SMILE is predictable, effective, and safe in correcting myopic anisometropia. The postoperative changes in HOAs are characteristic.

## Introduction

Anisometropia refers to the unequal refraction of two eyes. The mild differences in refraction do not cause clinical symptoms due to binocular fusion. However, obvious anisometropia may impair central fusion function, resulting in stereoscopic and contrast sensitivity dysfunction, asthenopia, and even strabismus or amblyopia (1). Anisometropia can be classified into physiological anisometropia and pathological anisometropia. Refractive differences of up to 1 diopter (D) are considered physiological (2), and more than 2.0 D are defined as pathological anisometropia (3).

Spectacles and contact lenses are the traditional treatment for anisometropia. With the advancement of refractive surgery, more and more people choose refractive surgery to correct anisometropia. Many studies have demonstrated that corneal refractive surgeries treat anisometropia safely and effectively. Common refractive surgeries include photorefractive keratectomy (PRK), laser *in situ* keratomileusis (LASIK), laser-assisted subepithelial keratectomy (LASEK), and small incision lenticule extraction (SMILE) (4–11). SMILE surgery is preferred for patients with anisometropia due to its safety, efficacy, predictability, and fewer valve-related complications (12–16).

Changed corneal morphology and higher-order aberrations (HOAs) after SMILE have been reported (17–19). However, it was rarely reported whether the corneal morphological changes after SMILE are characteristic in the two eyes of a patient with anisometropia. Therefore, this study investigated the corneal morphological changes in adult patients with myopic anisometropia after SMILE and the safety, efficacy, and predictability.

## Patients and methods

This was a prospective cohort study. Patients with myopic anisometropia were included in this study who underwent SMILE at the Ophthalmology Department of the First Affiliated Hospital of Chongqing Medical University from September 2019 to March 2021. The study was conducted in compliance with the principles of the Declaration of Helsinki and approved by the Ethics Committee of the First Affiliated Hospital of Chongqing Medical University. Written informed consent for surgery was obtained from patients and their immediate family members.

### Inclusion criteria

(1) Age ≥18 years; (2) binocular myopic anisometropia [spherical equivalent (SE) >2.0 D]; (3) corrected cylinder power ranging from −9 D to −0.25 D; (4) the best-corrected distance visual acuities (CDVA) in both eyes ≥20/25; (5) stable refractive status ≥2 years; and (6) normal corneal topography.

### Exclusion criteria

(1) Corrected distance visual acuity in one or both eyes <20/25; (2) patients diagnosed with other ocular and systemic diseases or more likely to scar; (3) history of ocular surgery; (4) patients with severe dry eye or other cornea diseases; and (5) pregnant or lactating women.

### Surgical methods

All the patients were administered levofloxacin eye drops (5 ml: 24.4 mg, Cravit^®^, Santen, Osaka, Japan) and sodium hyaluronate eye drops (10 ml: 0.1%, Hylo-comod^®^, RSAPHARM Arzneimittel GmbH, Industriestraße, Saarbrücken, Germany) four times a day, 3 days before the surgery. Intraoperatively, oxybuprocaine hydrochloride eye drops (20 ml: 80 mg, Benoxil^®^, Santen, Osaka, Japan) were administered twice for topical anesthesia. All SMILE surgeries were conducted using a VisuMax femtosecond laser system (Carl Zeiss Meditec AG, Jena, Germany). The femtosecond laser parameters were set as follows: pulse frequency, 500 kHz; pulse energy, 135 nJ; spot size, 4.3 μm; optical zone diameter, 6.0–6.7 mm; corneal cap diameter, 6.8–7.5 mm; cap thickness, 120 μm; incision length, 2.8 mm; position of incision, 90°; and the edge cutting angle, 90°. After the canning, a micro-separator was used to separate and lift the corneal cap’s edge and separate the lens’s front and back surfaces in turn. Then, micro tweezers were employed to remove the stromal lens from the small incision under the corneal cap, and the wholeness of the stromal lens was carefully checked. Extra water was absorbed by a sterile sponge, and the operation was completed.

### Postoperative treatment

Tobramycin and dexamethasone eye drops (TobraDex^®^, Novartis, Rijksweg, Puurs) were administered immediately after the operation four times at 10 min intervals. Subsequently, the following eye drops were required to be applied concomitantly: 0.5% levofloxacin eye drops, four times a day for 21 days; tobramycin dexamethasone eye drops, four times a day for 1 week, and then replaced with loteprednol eye drops (5 ml: 0.5%, Lotemax^®^, Bausch & Lomb Incorporated, Tampa, FL, United States), three times a day for 1 week, then twice a day for 1 week, and finally once a day for 1 week; sodium hyaluronate eye drops, four times a day for 1 month.

### Follow-up examinations

Before the operation, patients underwent a slip lamp examination for the anterior segment and a fundus examination using an indirect ophthalmoscope after dilatation of pupils using compound tropicamide eye drops (1 ml, Zhuobi’an^®^, Sinqi Pharmaceutical, Shenyang, China). Snellen chart was used to check the distance vision before and after the surgery; corneal topography was obtained by performing Pentacam^®^ AXL panoramic biometer (Oculus GmbH, Wetzlar, Germany) by an experienced clinician. Image acquisition was performed on an automatic mode. A total of three images were obtained for each eye to collect the total corneal aberration data with a 6 mm diameter. Images with data over 8 mm diameter and quality marked as “OK” were selected for the data processing. Images with data equal to or less than 8 mm diameter or failed quality tests were excluded. Higher order aberration (HOA), coma aberration (Coma), trefoil aberration (Trefoil), and spherical aberration (SA) were analyzed by the root mean square (RMS, μm). Furthermore, the dilated and small pupil tests were conducted by the same experienced optometrist. SE was obtained by adding the sum of the sphere power with half of the cylinder power. Follow-up examinations were conducted at 1 week, 1 month, 3 months, and 6 months after the surgery, which included uncorrected distance visual acuity (UDVA), CDVA, diopters, effectiveness index (defined as preoperative CDVA/postoperative UDCA), safety index (defined as postoperative CDVA/preoperative CDVA), posterior corneal elevation (PCE), anterior and posterior corneal radius of curvature of the 3 mm area at the center of the thinnest point of the cornea (ARC and PRC), and the wavefront aberrations. A standardized pupil diameter of 6.0 mm was used to analyze aberration.

### Statistical analysis

SPSS 17.0 (IBM Corp., Armonk, NY, United States) software was used to analyze data. Data were presented as “mean ± standard deviation.” Paired *t*-test was applied to compare the two groups of continuous normally distributed variables. Pearson Chi-squared test was used for intra-group comparison. *P*-value < 0.05 was considered statistically significant.

## Results

### Baseline information

The study included 36 consecutive patients (mean age: 25.2 ± 6.4 years). Among them, 14 (38.9%) were males and 22 (61.1%) were females. For the two eyes of each patient, the one with higher myopia was defined as group A, and the fellow eye was group B. The preoperative refractive status and corneal thickness of both groups are presented in Table 1.

**TABLE 1:** Preoperative refractive and corneal data (mean ± SD).

	Group A	Group B	*P*
CCT (μm)	546.08 ± 23.11	546.08 ± 22.71	1.000
SE (D)	−6.45 ± 1.25	−3.76 ± 1.29	0.000
Sphere (D)	−6.00 ± 1.24	−3.22 ± 1.3	0.000
Cylinder (D)	−0.90 ± 0.73	−1.07 ± 0.71	0.168
Optical zone diameter (μm)	120.94 ± 8.89	127.50 ± 7.32	0.000
Cap thickness (mm)	6.46 ± 0.15	6.57 ± 0.63	0.000

SD, standard deviation; CCT, central corneal thickness; SE, spherical equivalent; D, diopter.

### Efficacy and safety

**FIGURE 1:**
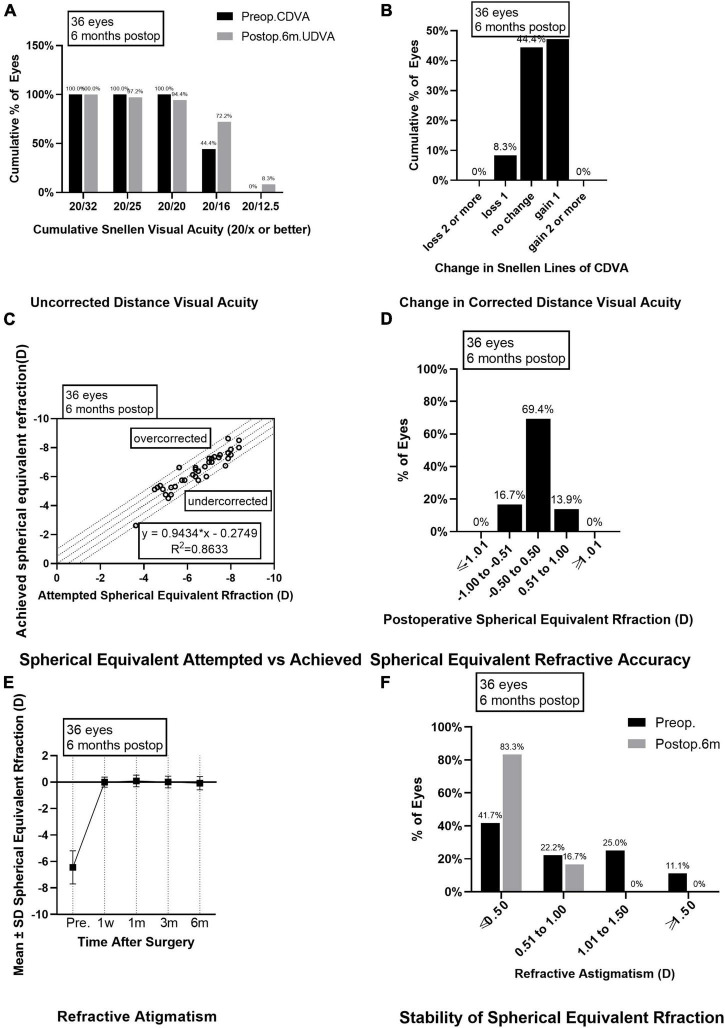
Outcomes of group A after SMILE surgery. **(A)** Cumulative percentage of preoperative CDVA and postoperative UDVA in group A at 6 months follow-up; **(B)** Change in CDVA in group A at 6 months follow-up; **(C)** Achieved versus attempted change in SE at 6 months follow-up in group A; **(D)** Accuracy of SE refraction in group A at 6 months follow-up; **(E)** Stability of SE in group A at 1 week, 1 month, 3 months, and 6 months after surgery; **(F)** The percentage of preoperative postoperative refractive astigmatism in group A at 6 months follow-up. CDVA, corrected distance visual acuity; UDVA, uncorrected distance visual acuity; SE, spherical equivalent.

**FIGURE 2:**
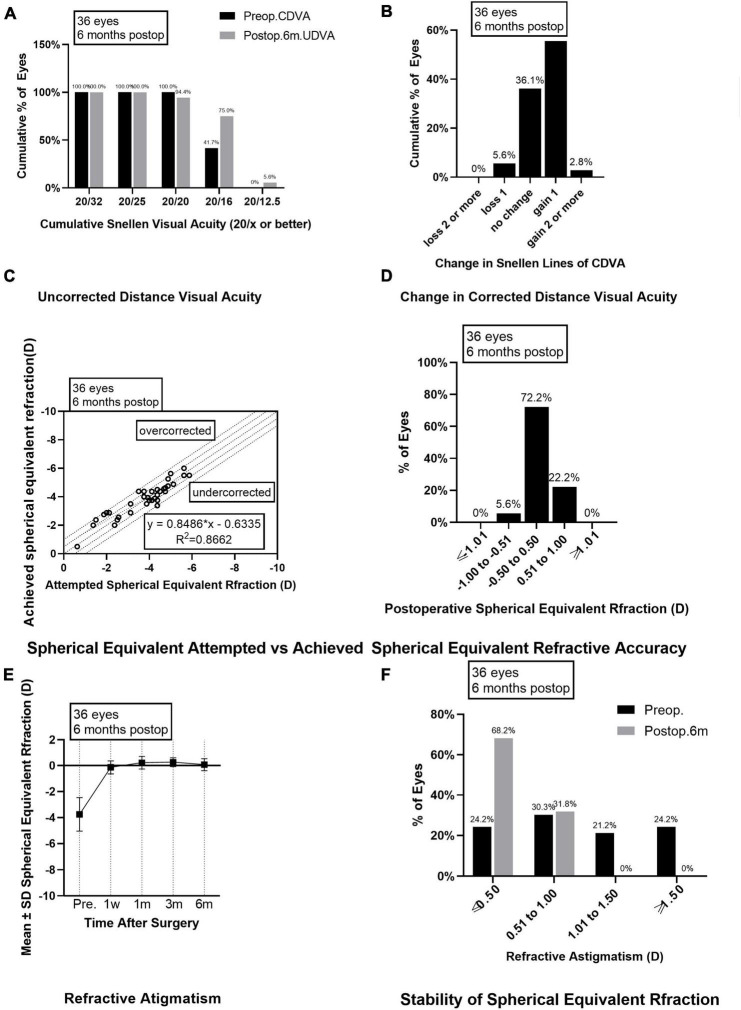
Outcomes of group B after SMILE surgery. **(A)** Cumulative percentage of preoperative CDVA and postoperative UDVA in group B at 6 months follow-up; **(B)** Change in CDVA in group B at 6 months follow-up; **(C)** Achieved versus attempted change in SE at 6 months follow-up in group B; **(D)** Accuracy of SE refraction in group B at 6 months follow-up; **(E)** Stability of SE in group B at 1 week, 1 month, 3 months and 6 months after surgery; **(F)** The percentage of preoperative postoperative refractive astigmatism in group B at 6 months follow-up. CDVA, corrected distance visual acuity; UDVA, uncorrected distance visual acuity; SE, spherical equivalent.

Preoperative CDVAs in groups A and B had no significant difference and were equal to or higher than 20/20. Six months after the surgery, the postoperative UDVAs were 20/32 or better in all the eyes from group A or group B. Among them, 34 eyes (94.4%) in group A and 34 eyes (94.4%) in group B had UDVAs of 20/20 or better, 26 eyes (72.2%) in group A and 27 eyes (75.0%) in group B had UDVAs of 20/16 or better (Figures 1A, 2A). The effectiveness indexes 6 months after surgery were 1.06 ± 0.16 in group A and 1.07 ± 0.14 in group B (*P* = 0.750).

As for the safety evaluation, the number of eyes with no change in CDVA was 16 (44.4%) in group A and 13 (36.1%) in group B; with one line improved in 17 eyes (47.2%) from group A and 20 eyes (55.6%) in group B; with two lines improved in none eyes from group A and one eye (2.8%) in group B (Figures 1B, 2B); with one line decreased in three eyes (8.3%) from group A and two eyes (5.6%) from group B; none of the eyes in groups A and B lost two or more lines when compared the postoperative CDVAs to the preoperative CDVAs. The safety indexes were 1.08 ± 0.14 in group A and 1.12 ± 0.15 in group B (*P* = 0.173).

### Predictability and stability

Six months after the surgery, the linear regression equation of achieved SE after SMILE and attempted SE before SMILE was *y* = 0.9434 × *x*-0.2749 (*R*^2^ = 0.8633, *P* < 0.0001) in group A and *y* = 0.8486 × *x*-0.6355 (*R*^2^ = 0.8662, *P* < 0.0001) in group B, without difference between the two groups (*P* > 0.0001, Figures 1C, 2C). The SE correction errors were less than ± 0.5 D in 25 eyes (69.4%) in group A and 26 eyes (72.2%) in group B (Figures 1D, 2D). The mean SE refraction changes over time after SMILE in the two groups are presented in Figures 1E, 2E. There was a significant reduction in the mean SEs at 1 week, 1 month, 3 months, and 6 months after the surgery compared with the preoperative SEs (*P* < 0.05). However, there was no significant change in SEs from 1 week to 6 months after surgery in both groups A and B. Three months after surgery, SE in group B was significantly higher than in group A (*P* = 0.003). The mean SEs at 6 months after surgery were −0.09 ± 0.50 in group A and 0.07 ± 0.47 in group B, without a significant difference between them (*P* = 0.059).

### Astigmatism

The preoperative astigmatism was less than 0.5 D in 15 eyes (41.7%) in group A and 10 eyes (27.8%) in group B. The numbers and percentages of eyes with different levels of postoperative astigmatism at 6 months after surgery were as follows: (1) 0.00–0.50 D, 30 eyes (83.3%) in group A and 28 eyes (77.8%) in group B; (2) 0.50–1.00 D, 6 eyes (16.7%) in group A and 8 eyes (22.2%) in group B; (3) 1.00 D–1.50 D and >1.50 D, none eyes in groups A and B (Figures 1F, 2F).

### Corneal morphology

**FIGURE 3:**
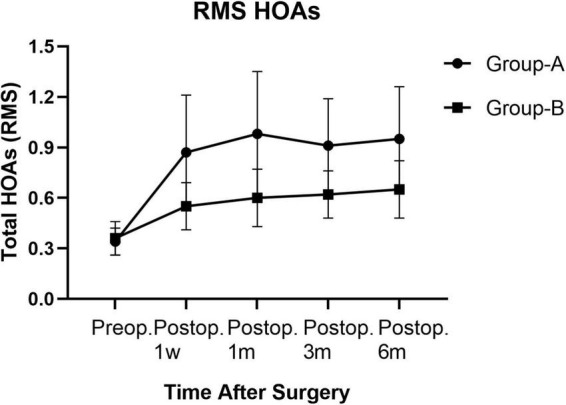
Root mean square HOAs of groups A and B from preoperative baseline to the 6 months after surgery.

Posterior corneal elevation: There was no significant difference in PCE between group A and group B before SMILE or at 1 week, 1 month, 3 months, and 6 months after SMILE. In groups A and B, compared to the preoperative PCE, there was no significant change in PCE at 1 week, 1 month, and 3 months after surgery (*P* > 0.05). However, it significantly decreased 6 months after surgery (*P* < 0.05). There was no significant change in PCE from 1 week to 6 months after surgery in groups A and B (*P* > 0.05, Table 2). ARC: There was no significant difference in ARC between group A (7.76 ± 0.22) and group B (7.77 ± 0.22) preoperatively. The ARC was significantly increased postoperatively than before the surgery in the two groups (*P* < 0.000). The ARCs were higher in group A than in group B at 1 week, 1 month, 3 months, and 6 months after surgery (*P* = 0.000, Table 3). PRC: There was no significant difference in PRC between group A and group B preoperatively and postoperatively at 1 week, 1 month, 3 months, and 6 months. PRCs did not change significantly after the surgery compared to the preoperative values in the two groups (*P* > 0.05). HOAs: As is seen in Table 4, before surgery, the root means square (RMS) value was 0.34 ± 0.08 in group A and 0.36 ± 0.10 in group B (*P* = 0.063). RMS values increased after SMILE in both groups compared to those before surgery (*P* < 0.05). As is seen in Figure 3, the postoperative RMS value in group A was significantly higher than in group B (*P* = 0.000). Compared to the values before surgery, there were significant increases in postoperative SA in groups A and B (*P* < 0.05). In both groups, coma 90° increased after surgery. The coma 90° value in group A was significantly higher than that in group B at 1 week, 1 month, 3 months and 6 months after surgery (*P* = 0.000). In both groups, there was no significant change in trefoil 0°, trefoil 30°, and coma 0° during the 6 months follow-up. For RMS values of total HOAs, SA, and coma, no significant changes were found between 1 week and 6 months postoperatively (*P* > 0.05). At 6 months postoperatively, there was no significant correlation between CDVA and HOAs in group A (*r* = 0.002, *P* = 0.990). Moreover, similar results were found in group B (*r* = −0.313, *P* = 0.076).

**TABLE 2:** Posterior corneal elevation (PCE) in groups A and B.

	*n*	Group A	Group B	*P*
Preoperative	36	4.81 ± 3.30	5.11 ± 3.07	0.536
1 week post-op.	32	2.66 ± 2.88	3.59 ± 3.17	0.070
1 month post-op.	33	2.82 ± 4.77	3.12 ± 3.00	0.617
3 months post-op.	29	2.41 ± 2.54	2.97 ± 3.65	0.203
6 months post-op.	36	2.06 ± 2.61	2.39 ± 2.58	0.314
P	–	<0.05*	<0.05*	–

Post-op,: postoperative. *Six months post-op. vs. preoperative.

**TABLE 3:** Anterior corneal radius of curvature of the 3 mm area at the center of the thinnest point of the cornea (ARC).

	*n*	Group A	Group B	*P*
Preoperative	36	7.76 ± 0.22	7.77 ± 0.22	0.683
1 week post-op.	32	8.84 ± 0.37	8.41 ± 0.31	0.000
1 month post-op.	33	8.83 ± 0.35	8.44 ± 0.31	0.000
3 months post-op.	29	8.79 ± 0.36	8.42 ± 0.31	0.000
6 months post-op.	36	8.76 ± 0.36	8.40 ± 0.31	0.000
*P*		0.000*	0.000*	

Post-op, postoperative. *Six months post-op. vs. preoperative.

**TABLE 4:** Comparison of wavefront aberrations between groups.

	Preop. (*n* = 36)			Postop. 1 week (*n* = 32)			Postop. 1 month (*n* = 33)			Postop. 3 month (*n* = 29)			Postop. 6 month (*n* = 24)		
					
	Group A	Group B	*P*	Group A	Group B	*P*	Group A	Group B	*P*	Group A	Group B	*P*	Group A	Group B	*P*
RMS HOA	0.34 ± 0.08	0.36 ± 0.10	0.063	0.87 ± 0.34*	0.55 ± 0.14*	0.000^#^	0.98 ± 0.37*	0.60 ± 0.17*	0.000^#^	0.91 ± 0.28*	0.62 ± 0.14*	0.000^#^	0.95 ± 0.31*	0.65 ± 0.17*	0.000^#^
Spherical aberration	0.16 ± 0.08	0.16 ± 0.07	0.692	0.44 ± 0.21*	0.20 ± 0.14	0.000^#^	0.44 ± 0.19*	0.22 ± 0.12	0.000^#^	0.36 ± 0.17*	0.20 ± 0.16	0.000^#^	0.38 ± 0.15*	0.21 ± 0.14	0.000^#^
Trefoil 30°	−0.04 ± 0.11	−0.07 ± 0.13	0.121	−0.03 ± 0.12	−0.01 ± 0.13	0.524	−0.01 ± 0.14	−0.07 ± 0.13	0.115	−0.05 ± 0.17	−0.09 ± 0.15	0.253	−0.07 ± 0.15	−0.05 ± 0.17	0.537
Coma 90°	−0.03 ± 0.15	−0.02 ± 0.17	0.647	−0.34 ± 0.42*	−0.21 ± 0.23*	0.035^#^	−0.42 ± 0.53*	−0.23 ± 0.27*	0.027^#^	−0.43 ± 0.42*	−0.21 ± 0.29*	0.001^#^	−0.34 ± 0.48*	−0.23 ± 0.28*	0.026^#^
Coma 0°	−0.03 ± 0.13	0.03 ± 0.14	0.128	−0.10 ± 0.39	−0.05 ± 0.22	0.532	−0.17 ± 0.38	−0.03 ± 0.26	0.151	−0.18 ± 0.43	−0.05 ± 0.29	0.289	−0.20 ± 0.39	−0.04 ± 0.27	0.072
Trefoil 0°	−0.02 ± 0.09	0.03 ± 0.11	0.055	−0.04 ± 0.21	0.05 ± 0.15	0.093	−0.05 ± 0.21	0.07 ± 0.15	0.039^#^	0.04 ± 0.20	0.06 ± 0.18	0.759	−0.04 ± 0.28	0.05 ± 0.21	0.236

*Significant difference in HOAs between preoperative and postoperative values. ^#^Significant difference in HOAs between Group A and Group B.

## Discussion

The results from the present study demonstrated that SMILE is an effective and safe procedure for correcting the refraction of myopic anisometropia. The predictability and stability of the achieved SE are also good and in agreement with the previous reports. Our data supported why SMILE has been selected as one of the mainstream procedures among the cornea stromal refractive surgeries correcting anisometropia. Even though characteristic changes of corneal morphology after SMILE have been investigated in many studies (20, 21), few revealed the characteristics and differences of these changes between the two eyes in a myopic anisometropia patient. In the present study, we provided information that should be useful to guide our practice in the future.

Small incision lenticule extraction procedure seems to have similar predictability, independent of the diopters of myopic correction (20, 22). No significant difference after SMILE was found in the efficacy index and safety index between high myopia and mild or moderate myopia from the previous studies (22–24). In the present study, there was no difference in the efficacy and safety indexes between the two groups, which is consistent with the results of the previous studies. However, it was previously shown in studies (25, 26) that the inflammatory response and refractive regression after SMILE were more severe in the high myopia eyes, which was related to the recovery of postoperative vision and might adversely affect the efficacy index.

Changes in PCE after SMILE have been reported that PCE tended to decrease as compared with that preoperatively, and this decline remained stable from 1 week to a 5 years follow-up period (27, 28). In the present study, changes in PCE in the higher myopia eyes were consistent with the previous studies in the higher and lower myopia eyes. This may be explained by the corneal reaction induced by laser surgery. However, a study on LASIK showed that eyes with higher preoperative refraction require more laser ablation, thus making them more prone to anterior corneal shift (29). It should be noted that although there was a tendency for posterior corneal surface elevation in the high myopia group, no iatrogenic keratectasia was observed in the present study.

Higher-order aberration changes after SMILE and is related to the visual quality. Induction of spherical aberration is a typical side effect in the refractive surgery of myopia (30). The increase of coma may be related to laser energy density and the laser inclination angle during peripheral corneal ablation, the number of corrected diopters, the position of cap rim cut, and the decentration ablation (20). In the present study, RMS values of total HOAs changed after SMILE. SA and coma 90° increased significantly after surgery; while coma 0°, trefoil 0°, and trefoil 30° did not change significantly. HOAs also presented stable without changes during follow-up from 1 week to 6 months. The eyes with higher myopia had significantly higher postoperative HOA, SA, and coma 90° than those of the fellow eyes. Our results were consistent with some previous studies (31), but not all. For example, Jin et al. (20) reported that RMS values and SA in the high myopia group were higher than in mild to moderate myopia groups, but this difference did not appear in coma 90°. Zhao et al. (32) found that RMS, coma 90°, and SA of the high and mild myopia groups increased significantly after surgery. However, there was no significant difference between the two groups in the RMS values of total HOAs and specific HOAs. It has been reported that RMS values and SA significantly increased after surgery, decreasing from 3 months to 3 years postoperatively. However, Coma 90° increased and remained stable over 3 years postoperatively (18). Future studies are warranted to address the different results between those studies and the present study.

There are differences in postoperative corneal aberration changes caused by different surgical methods. Wu et al. (33) suggested that the SMILE procedure induced similar optical changes with FS-LASIK. Chen et al. (34) also found no significant difference in trefoil, horizontal coma, SA, and total HOA postoperatively between SMILE and wavefront-guided (WFG) FS-LASIK. However, higher vertical coma was shown in SMILE than in WFG FS-LASIK, which may be related to the difference in the location of the two surgical incisions. However, in other studies (35), at 3 months postoperatively, the average values for SA and horizontal coma were lower in the SMILE group compared with the FS-LASIK group. Yu et al. (36) reported that the changes in a vertical coma, SA, and HOA were significantly lower in SMILE than in LASEK 3 years after surgery. In conclusion, the magnitudes of the changes of HOAs after different surgical methods are still inconclusive, which may be related to the size of the optical zone (33), follow-up period, ethnic difference, corneal thickness, degree of myopia, and tear film stability. (18) Studies are warranted to answer the question.

In general, the present study had some limitations. The sample size of this study was not large enough, and the follow-up period could be longer. A larger sample size and more extended observation after SMILE are warranted in the future. The correlation between the HOAs after SMILE and the objective and subjective visual quality should also be evaluated.

## Conclusion

From the present study’s results, SMILE was safe, effective, predictable and stable in treating adult myopic anisometropia. The postoperative corneal morphological changes in HOAs were characteristic, i.e., RMS values, SA, and coma 90° increased significantly after SMILE and remained stable during the 6 months follow-up. The eyes with higher myopia had significantly higher postoperative HOA, SA, and coma 90° than those of the fellow eyes.

## Data availability statement

The raw data supporting the conclusions of this article will be made available by the authors, without undue reservation.

## Author contributions

All authors listed have made a substantial, direct, and intellectual contribution to the work, and approved it for publication.
